# Intra-Host Diversity of Dengue Virus in Mosquito Vectors

**DOI:** 10.3389/fcimb.2022.888804

**Published:** 2022-06-22

**Authors:** Amanda Makha Bifani, Tanamas Siriphanitchakorn, Milly M. Choy

**Affiliations:** Programme in Emerging Infectious Diseases, Duke-National University of Singapore (NUS) Medical School, Singapore, Singapore

**Keywords:** quasispecies, DENV, dengue, genome diversity, Aedes

## Abstract

Dengue virus (DENV) is the most common arbovirus, causing a significant burden on both the economy and global healthcare systems. The virus is transmitted by *Aedes* species of mosquitoes as a swarm of closely related virus genomes, collectively referred to as a quasispecies. The level of genomic diversity within this quasispecies varies as DENV moves through various ecological niches within its transmission cycle. Here, the factors that influence the level of DENV quasispecies diversity during the course of infection in the mosquito vectors are reviewed.

## Introduction

Dengue virus (DENV) is the most common insect-borne virus, that results in a myriad of disease presentation ranging from subclinical infection to dengue hemorrhagic fever and dengue shock syndrome. DENV imposes a significant burden globally, infecting an estimated 390 million people (Bhatt et al., 2013) and costing approximately 2.1 billion dollars (Beatty et al., 2011) annually. With increasing globalization, urbanization and a growing population, these numbers are estimated to increase considerably in the coming years (Gubler, 2011; Messina et al., 2019).

**Figure 1:**
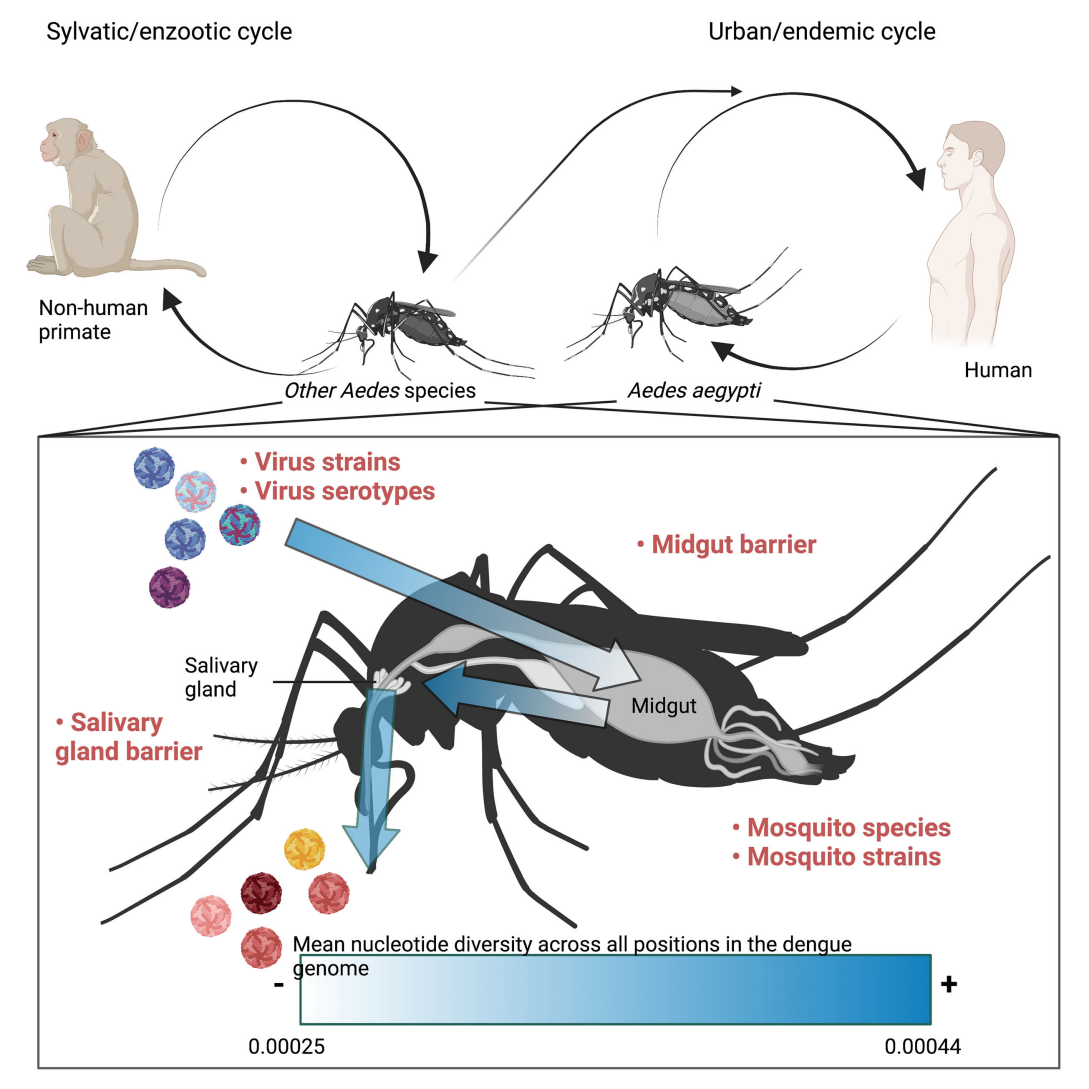
Schematic depicting dengue transmission among non-human primates and *Aedes* species in the sylvatic cycle and among humans and *Aedes aegypti* in the urban cycle as well as the transmission between the two cycles. The immune/genome diversity barriers imposed by anatomical features of *Aedes* mosquito species are further depicted. Arrows highlight the path of infection taken by DENV following an infected blood meal. Factors that influence the diversity of a quasispecies are written in bold red text. Arrows are shaded darker in areas where the mean nucleotide diversity across the DENV genome is higher whereas the lighter shade represents anatomical regions that are known to impose infection/genome diversity barriers and have a lower mean nucleotide diversity as determined by Lequime et al., 2016 (Lequime et al., 2016). The image was composed using Biorender.

DENV is an arbovirus, transmitted predominately by the mosquito vector, *Ae. (Aedes) aegypti* (Gubler, 1998). However, other *Aedes* species, such as *Ae. albopictus* can act as secondary vectors. Dengue is usually transmitted in an urban cycle between human and the *Ae. aegypti* mosquitoes (Weaver and Vasilakis, 2009) (**Figure 1**). However, a sylvatic cycle, in which the virus is passed between a non-human primate host and other *Ae.* species exists in forested areas (Weaver and Vasilakis, 2009) (**Figure 1**). Viruses found in the sylvatic cycle can be transmitted to urban cycles and vice versa (Pickering et al., 2020), leading to co-circulation of both urban and sylvatic DENV strains (Johari et al., 2019). To add further complexity to different genotypes of DENV that can be found within a single region, there are four antigenically distinct DENV serotypes (DENV1-4). Infection with a single serotype engenders protective immunity towards that serotype, but fails to protect against subsequent infection with a heterotypic serotype and may even lead to an enhanced disease phenotype through antibody dependent enhancement (Chan et al., 2019), making simultaneous transmission of different serotypes within the same region a public health concern.

DENV is a positive-sense single-strand RNA virus. The viral RNA is translated to a single polyprotein, which is subsequently cleaved into protein subunits by host and viral proteases (Guzman et al., 2016). The DENV proteome includes three structural proteins (Capsid; C, pre-Membrane; prM and Envelope; E) and seven non-structural (NS) (NS1, NS2A, NS2B, NS3, NS4A, NS4B, NS5) proteins. These proteins interact with one another as well as host factors to ensure successful infection and viral replication in the host cell. The genomic RNA is copied by the RNA-dependent RNA polymerase (RdRp) in the viral replication complex (Lescar et al., 2018). RNA viruses are notoriously error prone due to the lack of proof reading capabilities of the RdRp (Poirier and Vignuzzi, 2017). The DENV RdRp is estimated to carry an error rate of 1 in 10^4^ base pairs (bp) (Castro et al., 2005; Jin et al., 2011). In a genome of approximately 10,700 bp, this roughly translates to one error per dengue genome. As a result, DENV exists as a collection of closely related genomes, termed a quasispecies (Wang et al., 2002).

## Optimal Genome Diversity Level Required for Dengue Virus Fitness

Intra genetic diversity of a quasispecies has been known to impact the fitness, transmissibility as well as pathogenesis of a virus during infection (Lauring and Andino, 2010). Furthermore, the level of intra-species diversity is dynamic, changing over time when the viral population is faced with selective pressures that can result in population bottlenecks. In the case of other RNA viruses such as polio virus, a mutation in its RdRp conferred increased fidelity (Vignuzzi et al., 2006). This limited the virus’ ability to adapt to novel niches, ultimately resulting in a loss of neurotropism and pathogenesis (Vignuzzi et al., 2006; Vignuzzi et al., 2008). Increased polymerase fidelity has also been observed to attenuate human immunodeficiency virus (HIV) (Dapp et al., 2013; Beck et al., 2014; Beck et al., 2018; Davis et al., 2019). However, the converse is also capable of inducing an attenuated phenotype *in vivo.* When a mutator strain of SARS-CoV which lacked the exonuclease and therefore the virus’ proof-reading capabilities, was used to infect a mouse model, there was a reduction in pathogenesis (Graham et al., 2012). Similarly, mutator strains of coxsackievirus B3 and HIV have attenuated phenotypes compared to their parental strains (Gnadig et al., 2012; Dapp et al., 2013; Rozen-Gagnon et al., 2014). Likewise, for DENV and other closely related arboviruses, an optimal level of diversity in a quasispecies is required to maintain viral fitness (Vignuzzi et al., 2006; Beck et al., 2014; Rozen-Gagnon et al., 2014; Beck et al., 2018). Studies have demonstrated that strains that have a significantly elevated or reduced level of quasispecies diversity compared to wildtype tend to be less fit (Vignuzzi et al., 2008; Bifani et al., 2021). In DENV, clinically-tested successful attenuated vaccine candidate strains harbored a greater level of quasispecies diversity than their virulent parental strains (Bifani et al., 2021).

Genome diversity in arboviruses is particularly interesting as these viruses need to adapt to survive in at least two profoundly different environments, arthropod vector(s) and vertebrate host(s). Many arboviruses can infect a range of vertebrate hosts and mosquito vectors, which requires a flexible evolutionary strategy that would benefit from high mutation rate (Coffey et al., 2013). Indeed, an anti-mutator strain of Chikungunya virus (CHIKV) was found to decrease pathogenesis and transmissibility *in vivo* (Carrau et al., 2019). Related flavivirus, YFV (yellow fever virus) vaccine strains 17D and FNV have less quasispecies diversity than their respective parental strains, Asibi and FVV (Beck et al., 2014; Beck et al., 2018; Davis et al., 2019). It has also been suggested that the genomic diversity observed in West Nile virus (WNV) is engendered during the vector phase of the transmission cycle (Jerzak et al., 2007; Ciota et al., 2007). Meanwhile, there is evidence that both vector and human hosts drive DENV diversity (Lin et al., 2004; Sim et al., 2015; Sessions et al., 2015; Sim and Hibberd, 2016). Studies comparing DENV quasispecies diversity from infected human sera paired with mosquitoes infected from the patients’ sera revealed changes in the single nucleotide variants (SNVs) repertoire when transmitting from human host to mosquito vector (Sessions et al., 2015; Sim et al., 2015). Certain regions of the genome were more susceptible to genetic changes than others during this transition (Sessions et al., 2015). However, the quasispecies genome diversity is maintained through genetic bottlenecks and the introduction of novel single nucleotide variants (SNVs) (Sim et al., 2015). Previous studies have focused on the host’s contribution to DENV genomic diversity (Lin et al., 2004). However, contribution of the mosquito vector to xxx quasispecies maintenance is an engaging question which warrants further investigation.

As the level of quasispecies diversity oscillates when DENV progresses through different niches within the transmission cycles, the outcome of DENV infection is varied between humans and the mosquito vector. Whilst DENV infection is eventually cleared by the immune response of the human host, infection in the mosquito vector persists throughout the lifespan of the mosquito (Guzman et al., 2016). During the course of infection, various immune barriers in the mosquito vector, such as the midgut and the salivary glands, have been noted to induce genetic bottlenecks in arbovirus genomes (Forrester et al., 2012; Forrester et al., 2014). Yet, the overall genetic diversity of a viral population in these flaviviruses is restored by the time the infection reaches the salivary glands (Brackney et al., 2011; Sim et al., 2015). Several questions remain to be addressed, such as what drives the recovery of genomic diversity of a quasispecies following bottleneck events and when this increase in diversity occurs.

## Infection Barriers That Shape Denv Genetic Diversity

During the life cycle of DENV in the mosquito vector, the virus encounters multiple organ/tissue barriers which can either restrict or drive the diversity of viral quasispecies (**Figure 1**). Among them, the barriers associated with the midgut and the salivary glands are the most well-recognized (Lequime et al., 2016).

Upon ingestion of a DENV-infectious blood meal by a mosquito, the virus first establishes infection in the mosquito midgut. This stage is commonly referred to as the midgut infection barrier (MIB) (Gubler, 2011). It was reported that this initial midgut infection represents the first bottleneck on the incoming viral population, potentially through the mosquito immunity in the midgut epithelial cells (Lequime et al., 2016)(**Figure 1**). The study using DENV1 to infect four different mosquito genotypes suggests that the midgut bottleneck is severe, leading to a founder’s effect of about five to forty-two DENV1 genomes for each genotype respectively (Lequime et al., 2016). The virus population bottleneck that occurs at this stage appears to be a stochastic process (Ciota et al., 2012; Lequime et al., 2016). Following the midgut infection, DENV encounters the midgut escape barrier (MEB) during its dissemination from the midgut into the haemocoel (Gubler, 2011). This barrier represents a major bottleneck during DENV infection in the mosquito; even with successful midgut infection, viruses could still be inefficiently disseminated into the haemocoel (Gubler, 1998). The mechanism by which this happens remains undefined, but it was believed to be associated with the midgut basal lamina as an anatomical barrier.

Viral dissemination from the midgut into the haemocoel allows DENV to access several secondary target organs/tissues throughout the mosquito body. Among them, the salivary glands are essential for virus transmission. DENV must infect the salivary glands (the salivary gland infection barrier; SGIB) and finally disseminate into the salivary duct (the salivary gland escape barrier; SGEB) to be secreted with the mosquito saliva during subsequent blood meals (Gubler, 2011). The SGIB as a second, albeit less selective, immune barrier represents a relatively small bottleneck compared to the MIB (Forrester et al., 2012). One plausible reason could be because of the earlier necessary passage through the basal lamina of the salivary glands. During dissemination from the salivary glands, the bottleneck of virus genome diversity has also been observed (Gubler, 1998). However, the level of the bottleneck at this stage during *in vivo* transmission requires further investigation as most of the current studies have been performed using *in vitro* saliva collection (Weaver and Vasilakis, 2009; Johari et al., 2019; Pickering et al., 2020).

Recovery of the quasispecies diversity, however, seems to be dependent on the mosquito genetics, suggesting that once the founder’s event has occurred, there is purifying selection for novel SNVs based on the mosquito response to infection (Lequime et al., 2016). While the number of viral SNVs were found to increase once the midgut barriers were circumvented, consensus changes were only found in the salivary glands (Lequime et al., 2016). This implies that the salivary glands act as a secondary selective barrier during viral infection in the mosquito. Indeed, the salivary glands have been observed to function as a selective pressure for arboviruses such as CHIKV (Forrester et al., 2012). Nevertheless, there is an increase in the mean nucleotide diversity (π) in the salivary glands compared to the midgut (Lequime et al., 2016) (**Figure 1**).

If the midgut and salivary gland barriers act to restrict DENV quasispecies diversity, what enables the virus population to restore this variation in the population? Studies with other flaviviruses hint that the vector immune response, in particular the RNAi machinery stimulates quasispecies diversity. The genomic diversity of WNV passaged in the absence of RNAi machinery was significantly less diverse than when the RNAi machinery was functional (Brackney et al., 2015). Furthermore, regions of the WNV that are targeted to a greater extent by RNAi contain a greater number of point mutations, indicating the presence of positive selection (Brackney et al., 2009). Whether the RNAi machinery of *Aedes* mosquitoes also plays a role in driving diversification on the DENV genome remains to be explored.

## The Role Of Different Mosquito Vectors in Denv Quasispecies Diversity

DENV is principally transmitted by *Ae. aegypti* in the urban transmission cycle and *Ae. albopictus* in a peri-rural setting (Guzman et al., 2016). Naturally, the question arises if the different vectors differentially influence the level of DENV quasispecies diversification. Indeed, when the genomic diversity of DENV1 was examined in both *Aedes* species, there were SNVs unique to *Ae. aegypti* and others specific to *Ae. albopictus* (Sessions et al., 2015). Furthermore, when strains with a recent history of spillover from sylvatic to urban cycles were compared to urban endemic strains, it was found that they did not infect *Ae. aegypti* as readily (Pickering et al., 2020). In a way, these results are not surprising as it is expected that vector competence would be increased for strains naturally occurring in their respective vectors. This has been supported by an observed codon bias in strains specifically adapted to each vector (Dos Passos Cunha et al., 2018). Nevertheless, viruses jumping between sylvatic and urban cycles give rise to the possibility of novel strains with epidemic or pathogenic potential to the human population.

## Future Directions

Previous studies have clearly demonstrated that quasispecies diversity has the ability to shape viral fitness and pathogenesis (Vignuzzi et al., 2006). Arbovirus strains that have significantly more or less population diversity than wildtype stains have been associated with diminished fitness (Coffey et al., 2011; Beck et al., 2014; Rozen-Gagnon et al., 2014; Beck et al., 2018; Bifani et al., 2021). However, alternating between a mammalian host and a mosquito vector imposes restrictions on diversity of the DENV genome which are lost when DENV is exclusively passaged in either mammalian or mosquito cells (Dolan et al., 2021). As a result, certain regions in the genome are more amenable to changes when switching between hosts, while others are more conserved following transmission events (Sessions et al., 2015). There is no doubt that DENV quasispecies diversity is influenced by both human and mosquito vector (Sim et al., 2015). Here, special focus is given to contribution of mosquito vectors in maintaining quasispecies diversity. While *in vivo* studies in mosquitoes on arbovirus quasispecies diversity have been fruitful, there is still a dearth of knowledge to understand how the genetic diversity shaped by the anatomical and immune barriers of mosquitoes can influence virus fitness. The *Ae. aegyp*ti and *Ae. albopictus* mosquito genomes remain poorly annotated, and heterogeneity in the mosquito genome exists between different mosquito strains/species that can influence viral genetic diversity and virus fitness (Black et al., 2002). Several questions remain and warrant further investigation (**Table 1**). Teasing apart which stage of the DENV life cycle in the mosquito vectors enhances and diminishes viral genetic diversity and how these virus populations influence virus fitness would be an interesting area of study. As various host cells impact viral mutation rates differently (Combe and Sanjuan, 2014), it is plausible that certain cell types within the mosquito vectors are able to recover the genetic diversity lost after passages through anatomical and immune barriers. Additionally, exploring whether there is a difference in the amount of genetic diversity induced by *Ae. aegypti* or *Ae. albopictus* could be beneficial in predicting strains with epidemic potential.

**Table 1:** DENV quasispecies diversity key questions?

DENV quasispecies diversity key questions
• Does quasispecies diversity influence the length of the extrinsic incubation period? • Are strains with higher levels of quasispecies diversity more or less likely to carry epidemic potential? • Does quasispecies diversity impact vector competence? • What maintains the level of quasispecies diversity in the mosquito? • Do *Aedes aegypti* or *Aedes albopictus* impact the level of quasispecies diversity differently? If so, which *Aedes* species has a greater contribution to quasispecies diversity?

However, it is noteworthy that a detailed characterization of the mosquito would be complicated by the presence of many different body compartments and tissues. Moreover, while the conventional sample pooling strategy adopted to achieve sufficient starting materials for sequencing has proved fruitful in identifying common variants, it may obscure low-frequency variants due to the averaging effect of combining individuals. Despite that, advances in next generation sequencing capabilities, specifically the ability to call low frequency variants (Wilm et al., 2012; Bifani et al., 2021) provide new opportunities to examine viral genome diversity at the population level rather than the consensus, and rare variants would likely be discovered more in the coming years (Dolan et al., 2021). Furthermore, novel technologies that allow for the manipulation of the viral quasispecies composition by targeting specific variants could aid in ascertaining the function of specific variants. One such approach is a CRISPR-Cas9 system using CRISPR RNA with a shorter spacer region that has been shown to effectively suppress DENV and the related flavivirus ZIKV (Zika virus) (Singsuksawat et al., 2021; Chen et al., 2022). Without a doubt, there is much to uncover about the dynamics of DENV quasispecies diversity and its influence on fitness as well as shaping the DENV strains of the future.

## Authors Contributions

AB contributed to the conceptualization, literature review, writing and editing of this manuscript. TS contributed to the literature review. MC contributed to the conceptualization, literature review and editing of this manuscript. All authors contributed to the article and approved the submitted version.

## Funding

This work was funded by the Open-Fund-Young Individual Research Grant administered by the National Medical Research Council of Singapore (MOH-000630 (MOH-OFYIRG20nov-0005)).

## Conflict of Interest

The authors declare that the research was conducted in the absence of any commercial or financial relationships that could be construed as a potential conflict of interest.

## Publisher’s Note

All claims expressed in this article are solely those of the authors and do not necessarily represent those of their affiliated organizations, or those of the publisher, the editors and the reviewers. Any product that may be evaluated in this article, or claim that may be made by its manufacturer, is not guaranteed or endorsed by the publisher.
